# Recommended Mass Spectrometry-Based Strategies to Identify Botulinum Neurotoxin-Containing Samples

**DOI:** 10.3390/toxins7051765

**Published:** 2015-05-19

**Authors:** Suzanne R. Kalb, Jakub Baudys, Dongxia Wang, John R. Barr

**Affiliations:** Centers for Disease Control and Prevention, 4770 Buford Hwy NE, Atlanta, GA 30341, USA; E-Mails: skalb@cdc.gov (S.R.K.); jbaudys@cdc.gov (J.B.); dov2@cdc.gov (D.W.)

**Keywords:** botulinum neurotoxin, botulism, mass spectrometry

## Abstract

Botulinum neurotoxins (BoNTs) cause the disease called botulism, which can be lethal. BoNTs are proteins secreted by some species of clostridia and are known to cause paralysis by interfering with nerve impulse transmission. Although the human lethal dose of BoNT is not accurately known, it is estimated to be between 0.1 μg to 70 μg, so it is important to enable detection of small amounts of these toxins. Our laboratory previously reported on the development of Endopep-MS, a mass-spectrometric‑based endopeptidase method to detect, differentiate, and quantify BoNT immunoaffinity purified from complex matrices. In this work, we describe the application of Endopep-MS for the analysis of thirteen blinded samples supplied as part of the EQuATox proficiency test. This method successfully identified the presence or absence of BoNT in all thirteen samples and was able to successfully differentiate the serotype of BoNT present in the samples, which included matrices such as buffer, milk, meat extract, and serum. Furthermore, the method yielded quantitative results which had z-scores in the range of −3 to +3 for quantification of BoNT/A containing samples. These results indicate that Endopep-MS is an excellent technique for detection, differentiation, and quantification of BoNT in complex matrices.

## 1. Introduction

Botulinum neurotoxins (BoNTs) are a family of highly toxic proteins produced by various species of clostridia. BoNTs cause the disease known as botulism, a potentially lethal disease if untreated. BoNTs are currently classified into seven confirmed serotypes (A through G), and serotypes /A, /B, /E, and /F are known to cause disease in humans. All BoNTs consist of a heavy chain (HC) which is responsible for receptor binding and aiding in translocation across the cell membrane, and a light chain (LC) which is a highly-specific protease targeting neuronal proteins. Botulism is characterized by descending flaccid paralysis caused by cleavage of one or more of the proteins that comprise the Soluble NSF Attachment Protein Receptor (SNARE) complex. BoNT/A, /C, and /E cleave SNAP (synaptosomal-associated protein)-25 [1,2,3,4,5,6] whereas BoNT/B, /D, /F, and /G cleave synaptobrevin-2 (also known as VAMP-2) [7,8,9,10,11,12]. BoNT/C is unique in that it is known to cleave more than one protein as it also cleaves syntaxin [13,14]. Additional variation within most serotypes exists and is designated as subtype, noted with a letter and a number, e.g., BoNT/A1 or BoNT/A2.

Previously, our laboratory reported on the development of the Endopep-MS method to detect BoNTs present in buffer [15]. This method is an *in vitro* activity assay, detecting the enzymatic activity of the LC of BoNT. Instead of examining the enzymatic activity of the LC on the toxin’s *in vivo* protein target, BoNT’s enzymatic activity upon a peptide substrate, which mimics the toxin’s *in vivo* protein target, is measured. Cleavage of the peptide substrate is observed using mass spectrometry, enabling the rapid detection of the exact location of the substrate cleavage by determining the mass of the cleavage products. Because the substrate cleavage location is serotype-specific, the Endopep-MS method allows for serotype differentiation. The addition of an immunoaffinity enrichment step before incubation with the peptide substrate has proven successful at detecting and differentiating BoNT in clinical specimens [16], culture supernatants [17], and foods [18]. The Endopep-MS method attains limits of detection similar to or below that of the historically used mouse bioassay [19].

In addition to the superb limits of detection, the assay is highly specific, with three layers of specificity. First, the toxin must bind to the correct antibody, e.g., BoNT/A must bind to antibodies specific for BoNT/A. The antibodies used in the assay are a mixture of monoclonal antibodies chosen for their high affinity to the BoNTs, with an affinity in the pM range [20,21,22] and bind to all known subtypes. Next, the toxin must retain adherence to the correct antibody in the presence of 2M NaCl, as the antibody-coated beads are washed in 2M NaCl after extraction of the BoNT from the sample matrix. This 2M NaCl wash immensely decreases the amount of non-specific binding to the antibodies. Finally, the toxin must also cleave at the correct protein site, which is detectable by mass spectrometry.

Because public health needs usually only require the detection of toxin and differentiation of serotype, the Endopep-MS assay is typically a qualitative assay. It can be converted into a quantitative assay, however, through the addition of an internal standard into all the samples and including a standard curve consisting of known amounts of BoNT spiked into the same matrix as the sample. Through comparison of the peak area of the cleavage products to the peak area of the internal standard, the level of toxin in the sample in question can be quantified, provided that the level of toxin in the sample falls within the toxin levels spiked into the standard curve. The Endopep-MS assay has effectively quantified BoNT serotypes which affect humans in clinical matrices [23] and culture supernatants [24].

Due to our previous success in detecting, differentiating, and quantifying BoNT in complex matrices, we decided to apply the Endopep-MS method to the analysis of thirteen blinded samples supplied as part of the EQuATox BoNT international proficiency test. The goal of this work was to first identify the presence or absence of BoNT in the blinded samples, consisting of buffer, milk, meat extract, and serum. Furthermore, for the samples which were found to contain BoNT, we sought to differentiate the serotype of BoNT and quantify the amount of toxin in the sample. Finally, where possible, we opted to extend the analyses further, obtaining additional information on the toxins to the subtype level and beyond through mass spectrometric amino acid sequencing.

## 2. Results

### 2.1. BoNT Detection and Differentiation

Because the Endopep-MS assay detects BoNT on the basis of peptide substrate cleavage and every serotype of BoNT has a different peptide substrate or cleaves the peptide substrate in a different location, the assay simultaneously differentiates the serotype of BoNT upon detection of BoNT. A 10 μL aliquot of each of the 1mL samples was selected for BoNT/A testing, and an additional 10 μL of each of the samples was allocated for BoNT/B, /E, and /F testing. Ten of the thirteen samples were found to be positive for BoNT/A, two samples were positive for BoNT/B, and one sample was positive for BoNT/E. Specifically, samples 1, 2, 5, 7, 8, 9, 10, 11, 12, and 13 were positive for BoNT/A, samples 6 and 8 were positive for BoNT/B, and sample 4 was positive for BoNT/E, as seen in Table 1.

**Figure 1:**
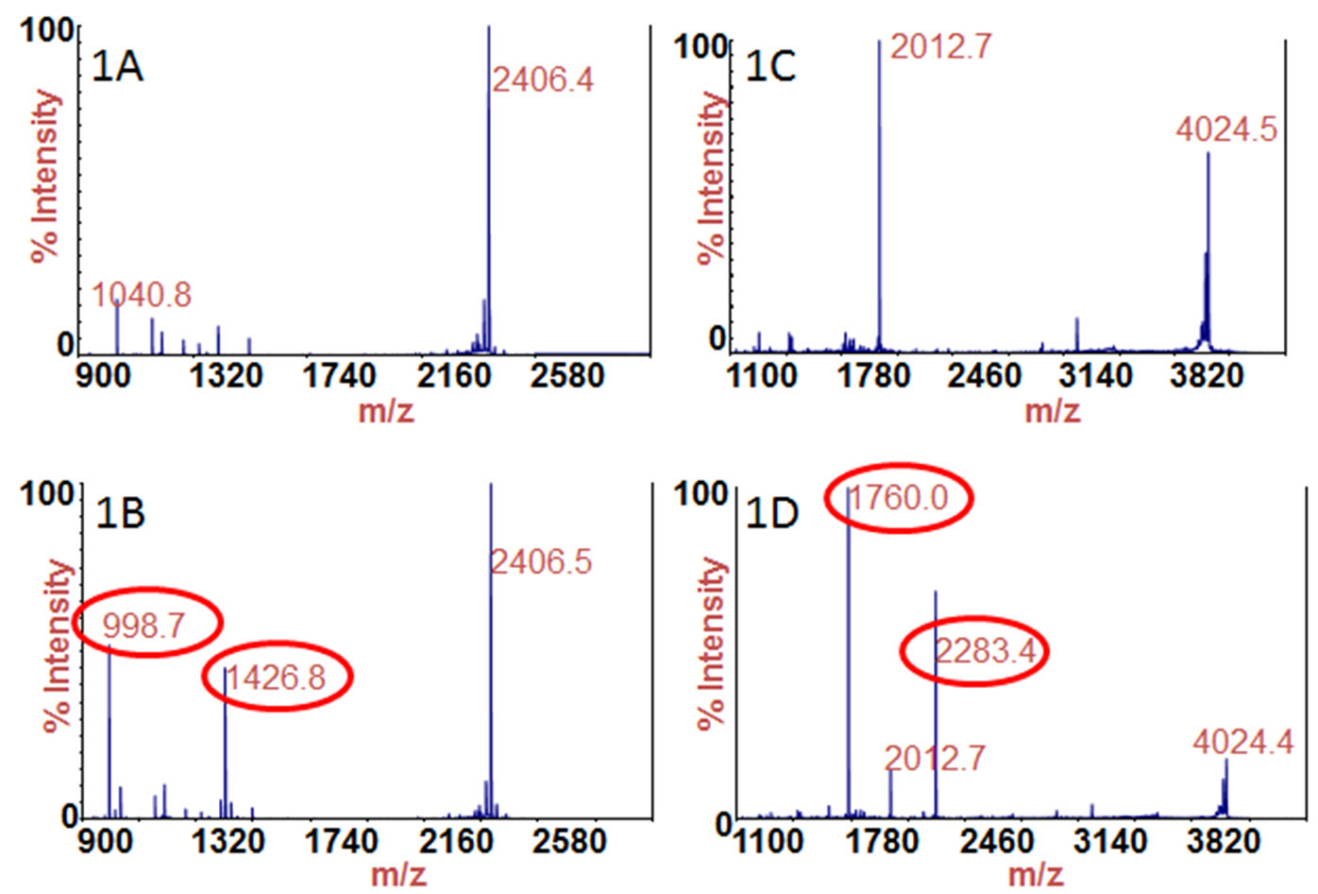
Mass spectra of the Endopep-MS reaction for (**A**) negative control tested for BoNT/A; (**B**) 10 μL of sample 8 tested for BoNT/A; (**C**) negative control tested for BoNT/B; and (**D**) 10 μL of sample 8 tested for BoNT/B, /E, and /F. The cleavage products of SubA or SubB indicating the presence of BoNT/A or /B are circled in red.

Sample 8 was a clear liquid which tested positive for BoNT/A, and after testing was revealed to be 0.1% BSA (bovine serum albumin) in PBS (phosphate buffered saline) with 4.7 ng/mL of BoNT/A. Figure 1A is the mass spectrum of the negative control of testing for BoNT/A. The peak at *m/z* 2406.5 corresponds to the intact substrate for BoNT/A (SubA). Figure 1B is the mass spectrum of the Endopep-MS reaction of 10 μL of sample 8 tested for BoNT/A, or the equivalent of 47 pg of BoNT/A spiked into 0.1% BSA/PBS. Figure 1B contains a peak corresponding to intact SubA at *m/z* 2406.5. SubA is cleaved by BoNT/A to produce an *N*-terminal cleavage product at *m/z* 1426.8 and a *C*-terminal cleavage product at *m/z* 998.7, and those peaks serve as evidence for the existence of BoNT/A. Both cleavage products are visible and circled in red in Figure 1B.

Sample 8 was also found to contain BoNT/B, and after testing was reported to contain 4.5 ng/mL of BoNT/B. Figure 1C is the mass spectrum of the negative control for BoNT/B testing. The intact substrate for BoNT/B (SubB) is present at *m/z* 4024.4 and the doubly charged intact substrate ion at 2012.7. Figure 1D is the mass spectrum of the Endopep-MS reaction of 10 μL of sample 8 tested for BoNT/B, /E, and /F. The peak at *m/z* 4024.4 in Figure 1D corresponds to intact SubB. In the presence of BoNT/B, SubB is cleaved to produce an *N*-terminal cleavage product at *m/z* 1760.0 and a *C*-terminal cleavage product at *m/z* 2283.4, so those peaks indicate the existence of BoNT/B. Both cleavage products are visible and circled in red in Figure 1D, indicating that sample 8 also contains BoNT/B.

**Table 1:** Qualitative results for analyses of the EQuATox samples for BoNT/A, /B, /E, and /F by Endopep-MS.

Sample	Matrix	BoNT/A observed?	BoNT/B observed?	BoNT/E observed?	BoNT/F observed?	Actual BoNT
S1	Meat extract	Yes	X	X	X	BoNT/A
S2	0.1% BSA/PBS	Yes	X	X	X	BoNT/A
S3	0.1% BSA/PBS	X	X	X	X	None
S4	0.1% BSA/PBS	X	X	Yes	X	BoNT/E
S5	Meat extract	Yes	X	X	X	BoNT/A
S6	0.1% BSA/PBS	X	Yes	X	X	BoNT/B
S7	0.1% BSA/PBS	Yes	X	X	X	BoNT/A
S8	0.1% BSA/PBS	Yes	Yes	X	X	BoNT/A BoNT/B
S9	0.1% BSA/PBS	Yes	X	X	X	BoNT/A
S10	Milk	Yes	X	X	X	BoNT/A
S11	Serum	Yes	X	X	X	BoNT/A
S12	0.1% BSA/PBS	Yes	X	X	X	BoNT/A
S13	Milk	Yes	X	X	X	BoNT/A

**Figure 2:**
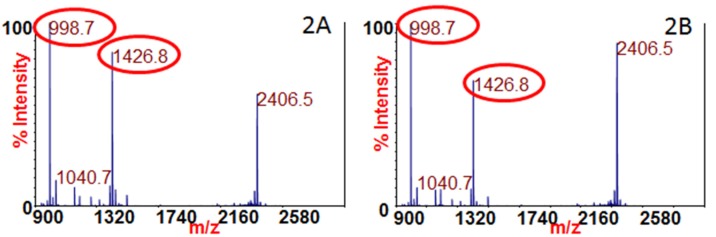
Mass spectra of 10 μL of samples tested for BoNT/A by Endopep-MS. (**A**) Sample 1 (meat extract) and (**B**) sample 11 (serum) both include the cleavage products of SubA indicating the presence of BoNT/A, circled in red.

Complex matrices in the EQuATox panel were also tested for BoNT/A, /B, /E, and /F. Samples 1 and 5 consisted of meat extract, samples 10 and 13 consisted of milk, and sample 11 consisted of serum. Because the Endopep-MS method uses antibody affinity to isolate the toxin from other proteins in the sample, meat extract, milk, and serum were not problematic for this assay. After the blind testing was concluded, sample 1 was revealed to be meat extract containing 10.5 ng/mL of BoNT/A. Figure 2A is the mass spectrum of the Endopep-MS reaction of 10 μL of sample 1, or the equivalent of 105 pg of BoNT/A in meat extract. The peaks at *m/z* 998.7 and 1426.8 are the cleavage products of SubA and provide evidence for the existence of BoNT/A in that sample. Similarly, those peaks are also present in Figure 2B, which is the mass spectrum of testing of 10 μL of sample 11, later revealed to be 9.8 ng/mL of BoNT/A in serum.

### 2.2. BoNT Quantification

**Figure 3:**
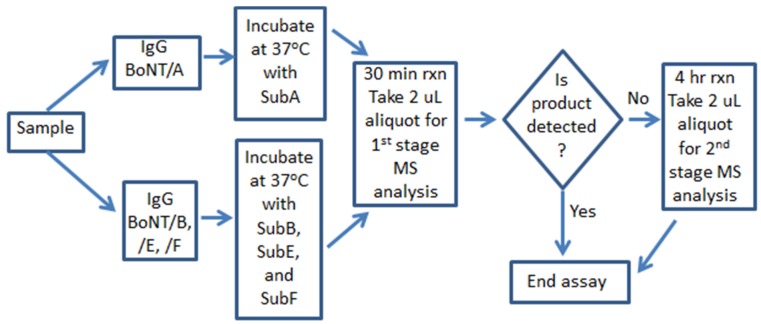
Schematic of steps in the Endopep-MS two-stage quantification for BoNT.

Through addition of an internal standard and the use of a calibration curve generated from matrix spiked with known amounts of BoNT/A, /B, /E, and /F, the Endopep-MS process can be made quantitative. The range of linearity was extended through the use of a two-stage quantification, with measurements performed after both 30 min and 4 h of incubation of the immuno-captured BoNT with the peptide substrate [24]. Figure 3 depicts the steps used in this dual-stage process. Our quantitative analyses of the 13 blinded samples were in agreement with our qualitative analyses, namely, BoNT/A was found to be present in 10 of the samples, BoNT/B was in two, and BoNT/E was in one sample. The levels of BoNT observed in the EQuATox samples ranged from 0.5 ng/mL to values in excess of 1000 ng/mL and are listed in Table 2, along with the nominal concentration of toxin spiked into the samples.

**Table 2:** Quantitative results of the Endopep-MS assay on the EquaTox samples for BoNT *.

Sample	Matrix	Serotype	Nominal Conc (ng/mL)	Obs Conc #1 (ng/mL)	Obs Conc #2 (ng/mL)	z-score
S1	Meat extract	BoNT/A	10.5	13	13	1.1
S2	0.1% BSA/PBS	BoNT/A	9.9	12	15	1.4
S3	0.1% BSA/PBS	None	N/A	<LOD	<LOD	N/A
S4	0.1% BSA/PBS	BoNT/E	10.9	23	20	3.8
S5	Meat extract	BoNT/A	108.0	170	180	2.4
S6	0.1% BSA/PBS	BoNT/B	9.0	19	18	4.1
S7	0.1% BSA/PBS	BoNT/A	100.0	120	130	1.0
S8A	0.1% BSA/PBS	BoNT/A	4.7	6.7	7	1.8
S8B	0.1% BSA/PBS	BoNT/B	4.5	6.2	6.1	1.4
S9	0.1% BSA/PBS	BoNT/A	0.5	0.56	0.65	0.9
S10	Milk	BoNT/A	10.3	14	14	1.4
S11	Serum	BoNT/A	9.8	16	15	2.3
S12	0.1% BSA/PBS	BoNT/A	1001.0	1800	1600	2.7
S13	Milk	BoNT/A	112.0	130	120	0.5

* Described in detail in: Qualitative and quantitative detection of BoNT from complex matrices: results of the first international proficiency test. S. Worbs, U. Fiebig, R. Zeleny, H. Schimmel, A. Rummel, W. Luginbühl, B. G. Dorner, manuscript in preparation.

**Figure 4:**
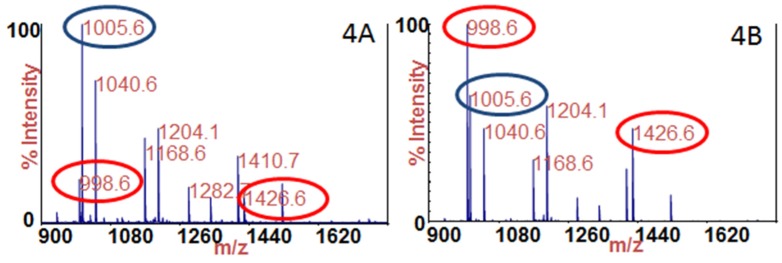
Mass spectra of the Endopep-MS reaction for 100 μL of sample 9 tested for BoNT/A after (**A**) 30 min and (**B**) 4 h of incubation with SubA. The cleavage products of SubA indicating the presence of BoNT/A are circled in red, and the internal standard is circled in blue.

Because the level of toxin in the samples differed by a range of greater than 3 orders of magnitude, the use of the two-stage quantification method assisted greatly in our ability to generate accurate values, as this two-stage method serves to extend the range of linearity. As an example, Figure 4 are the mass spectra of 100 μL of sample 9 tested for BoNT/A; Figure 4A was generated after incubation of only 30 min, whereas Figure 4B was generated after incubation of 4 h. The cleavage products indicating the presence of BoNT/A are circled in red, and the internal standard is circled in blue. By comparing the intensity of the cleavage products to the intensity of the internal standard, it is apparent that the intensity of both cleavage products increases with time. In addition to extending the dynamic range of the assay, use of the dual-stage method allowed for multiple measurements on the same sample, generating more accurate results, which is evidenced by the low z-scores for the BoNT/A measurements listed in Table 2.

### 2.3. BoNT Amino Acid Sequencing

**Figure 5:**
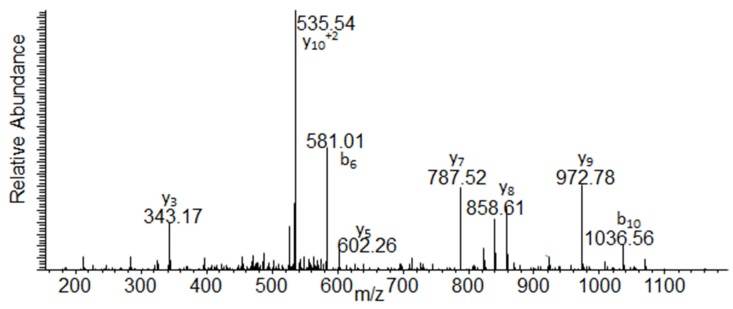
MS/MS of the peptide IPNAGQMQPVK from a tryptic digest of the immunocaptured BoNT/A from sample 12.

We obtained amino acid sequence information via LC-MS/MS on nine of the 13 samples. Samples 1, 2, 5, 7, 10, 11, 12, and 13 had sufficient toxin levels to discern the identity of the BoNT/A as the BoNT/A1 subtype, and the sequence coverages were 33.3%, 36.5%, 57.9%, 63.3%, 35.9%, 31.7%, 70.0%, and 49.6% respectively. The sequence coverage on samples 5, 7, and 12 was adequate to further differentiate the toxin as originating from the *C. botulinum* A1 Hall strain. Figure 5 is the MS/MS from the peptide IPNAGQMQPVK originating from the tryptic digest of the BoNT/A immunocaptured from sample 12. This peptide can be used to differentiate BoNT/A1 Hall from BoNT/A1(B) as the peptide originating from the A1(B) strain has the sequence IPNVGQMQPVK. Fragment ions b_6_, y_8_, y_9_, y_10_^+2^, b_10_ and the mass of the intact peptide are especially helpful for discerning these two closely related peptides as these include the alanine in position 4 which as a valine has a different mass in the peptide originating from the A1(B) strain.

## 3. Discussion

The Endopep-MS method successfully identified which EQuATox samples contained BoNT in all 13 samples. Although the Endopep-MS method can be used to detect all seven confirmed BoNT serotypes [25] and can also be used to detect BoNT/FA, formerly known as BoNT/H [26,27], analysis for this study only focused on detection of serotypes A, B, E, and F as these are the serotypes that primarily cause disease in humans, and participants in the project were told that only those four serotypes would be used for this study. In addition to the correct identification of samples containing BoNT, the Endopep-MS assay also correctly differentiated the serotype in all samples. Differentiation of the serotype occurs simultaneously with detection of the toxin, giving the Endopep-MS assay an advantage over some *in vivo* assays to detect BoNT, as many *in vivo* assays first identify the presence of the toxin and then identify the serotype through the use of serotype-specific antitoxins. EQuATox provided relatively low sample volumes, 1 mL, for all testing. At the concentrations of toxin provided in the proficiency test, the Endo-pep MS assay correctly identified the presence of toxin and the serotype using only 10 µL per sample. 

Additionally, the Endopep-MS method correctly identified the presence of two serotypes in one sample in the case of sample 8. Typically a sample contains only one serotype of toxin; however, some strains of *C. botulinum*, known as bivalent toxin producers, produce more than one serotype of BoNT. Our laboratory has proven successful at correct identification of the presence of both toxins from bivalent toxin producers [17], and was the first to report the discovery of three active BoNT produced from a trivalent toxin producer, *C. botulinum* Af84 [28]. Furthermore, the complex matrices used in this study, meat extract, serum, and milk, were not problematic for the Endopep-MS method as we successfully identified the correct serotype of BoNT in the presence of those matrices. The Endopep-MS method utilizes immunoaffinity to concentrate the toxin in the sample and remove the toxin from other proteins present in complex matrices that may interfere with the assay. In a protease-rich environment such as stool, there may still be some proteases which bind non-specifically to the antibodies, however immunoaffinity is just one aspect of specificity of the assay, and we have also implemented a stringent wash consisting of 2M NaCl to remove non-specific binding [29]. Utilizing antibodies with strong affinity for the toxin allows for stringent washes, increasing specificity of the assay.

The routine use of the Endopep-MS method on clinical and food samples is typically in a qualitative fashion because the detection of any BoNT is actionable and neither the response nor patient treatment are altered due to the amount of toxin detected in a sample. The Endopep-MS assay is semi-quantitative in its routine use as the ratio of the cleavage products to the intact substrate in the MALDI mass spectrum gives a quantitative estimate. For some studies and applications, a more quantitative measurement is beneficial and we have developed workflows to quantify the toxins. This include the addition of an isotopically labeled internal standard, a calibration curve, and detection by liquid chromatography tandem mass spectrometry [23] or more recently by MALDI TOF MS [24] Additionally, the use of two-stage quantification in the MALDI TOF MS method extends the dynamic range and has the potential to increase accuracy, allowing for two quantitative measurements on a single sample through the use of two time points [24]. 

Although for some applications, the weight of the toxin is a preferred measure, when BoNT is quantified for public health or pharmaceutical purposes, it is generally preferable to quantify the toxicity or potency of a sample because toxicity and potency are of greater relevance than the amount by weight (e.g., ng/mL). Thus, the unit generally used for BoNT quantification is mouse LD_50_ (mLD_50_), which is determined by carefully titering the toxin. An estimate of the total protein is also usually included but the total protein is a poor reflection on the actual amount of toxin present in most commercial standards because purity, di-chain toxin verses single chain toxin in complex, BoNT subtype, and quality of the toxin preparation all have a large impact on the relationship of total protein in a standard to the activity/toxicity of that standard. 

An additional problem with BoNT quantification is the lack of commutable reference materials to standardize or harmonize quantification of BoNT subtypes or even BoNT serotypes. Accurate BoNT quantification would require appropriate reference materials with known amounts of BoNT by weight and activity/toxicity and these standards would need to be commutable to a wide range of analytical techniques. Highly accurate and reproducible quantification by toxin weight also requires that both the serotype and subtype of BoNT in the sample to be known so that the appropriate subtype standards are employed for quantification. This is because toxin subtypes have different weight/activity ratios for activity/toxicity measures such as mouse bioassay, Endopep-MS, and mouse hemidiaphragm assays and also show differences in immunological assays such as ELISA [30]. 

EQuATox is the first international program to explore quantitative results for a variety of BoNT detection methods using a variety of analytical standards. The results from EQuATox will become an important basis to begin international discussions on what exactly are the needs for BoNT quantification for public health and counter terrorism and what reference standards and what level of standardization or harmonization are required to meet patient, public health and counter terrorism needs. Our laboratory obtained quantitative results for the EQuATox proficiency test. One measure of accuracy is a z-score, which indicates how many standard deviations a value is from the mean. z-scores in the range of −2 to +2 are considered to be acceptable, and seven of our ten BoNT/A measurements (Table 2) were within this range, and the remaining three BoNT/A z-scores were within the range of −3 to +3. No BoNT/A measurements were outside the range −3 to +3. 

Quantitative measurements of BoNT/B and /E however suffered from higher z-scores. One possible explanation is that the EQuATox program required that quantitative results should be reported in ng/mL and not in toxicity/potency units, although our quantification was performed in toxicity units of mLD_50_. So, the higher z-scores for the B and E serotypes could be due to differences in translating activity to weight from the commercially available standards used for our calibration as compared to the non-commercially available recombinant material that was used to spike the EQuATox samples. The quantification of BoNT/E in the EQuATox samples has even more complications that could affect the quantification and further explain the results as there are differences between the toxin used in the blinded samples and the toxin used in the calibration curve. The BoNT/E used in the EQuATox study was BoNT/E1 (GenBank ID of CAA43999) [31] whereas the BoNT/E used to create the calibration curve was BoNT/E3. An examination of the amino acid differences in the enzymatically-active light chain indicate that the light chain of BoNT/E1 and /E3 are only 94% identical. Such differences between toxins are known to cause differences in toxin activities for BoNT/A [32,33], so it possible that the higher z-score for our BoNT/E quantification is due to the difference in activities between the BoNT/E3 calibrant and the BoNT/E1 used in the study samples. 

One additional difference between the commercially available BoNT/E standards and the non-commercially available EQuATox material is in “activation” of the material. BoNT/E producing strains of *C. botulinum* do not produce the enzyme required to activate the toxin by cleaving the expressed single chain holotoxin species into a heavy and light chain. We designed a peptide substrate for BoNT/E for use in the Endopep-MS assay, which can be cleaved by the expressed single chain holotoxin or activated toxin. The commercially-available dichain BoNT/E toxin used for the calibration curves is not activated but the recombinant BoNT/E used by EQuATox was activated. While the Endopep-MS method can sensitively detect both expressed single chain holotoxin and activated BoNT/E, the relative enzymatic activity is likely different and therefore could add to the differences between the commercially available BoNT/E standards and the EQuATox material further affecting quantitative results. Such a discrepancy could be avoided through the use of standard calibrants, further highlighting the need for well-characterized calibrants for BoNT studies.

Differentiation of BoNT below the serotype level to the subtype/toxin variant level can be important for epidemiological or forensic purposes and can assist with the evaluation of potential antitoxin medical countermeasures. The primary structure of any protein is comprised of its amino acid sequence, so determination of the amino acid sequence allows for a high degree of differentiation between closely related proteins. Edman sequencing has traditionally been used for amino acid sequence identification, but more recently, mass spectrometry has proven to be a trustworthy technique for amino acid sequencing [34]. Provided that a sufficient level of BoNT is present in a sample, it is possible to differentiate the BoNT among proteins which are up to 99.8% identical, allowing for answers to epidemiologic or forensics questions. We were able to obtain sequence information on 9 of the 13 samples and identified the BoNT/A samples as BoNT/A1 Hall, indicating that at least 9 of the 13 samples were all spiked with the same BoNT/A.

## 4. Experimental Section

### 4.1. Materials

Monoclonal antibodies (mAbs) to BoNT/A (CR2 and RAZ1) [21], /B (2B18.2 and B12.1) [22], and /E and /F (6F5) [28] were obtained from Dr. James Marks at the University of California at San Francisco, and were used for immunoaffinity purification. Dynabeads (M-280/Streptavidin) were purchased from Invitrogen (Carlsbad, CA, USA). BoNT/A, /B, /E, and F dichain toxins at a concentration of 1 μg/mL were purchased from Metabiologics (Madison, WI, USA) and are the same standards typically used for the ELISA assays. Botulinum neurotoxin is very toxic and necessitates appropriate safety measures (see below). All chemicals were from Sigma-Aldrich (St. Louis, MO, USA) except where indicated. Peptide substrates for evaluation of BoNT activity are as reported previously [18] and were synthesized by Midwest Bio-tech Inc. (Fishers, IN, USA). Sulfo-NHS-Biotin was purchased from Thermo Fisher Scientific (Waltham, MA, USA). Kingfisher plates (deep well and traditional 96 well) and tip combs were purchased from Thermo Fisher Scientific (Waltham, MA, USA).

### 4.2. Preparation of mAb-Coated Beads

The mAbs were biotinylated and bound to streptavidin-coated beads using a previously described protocol [18].

### 4.3. Extraction and Incubation of BoNT

Biosafety Level-2 practices, processes, and facilities were used to ensure safety while working with BoNT. Additionally, toxin stock material and all samples containing BoNT were processed in a Class II biosafety cabinet containing HEPA filters to minimize the potential for aerosol exposure.

For qualitative analyses, 10 μL of each sample was added to 500 μL of phosphate buffered saline with tween (PBST) in duplicate. A negative control consisting of 500 μL of PBST and a positive control consisting of 500 μL of PBST spiked with either 1 mLD_50_ of BoNT/A or 1 mLD_50_ of BoNT/B, /E, and /F were run in parallel with the samples. Antibody coated beads were then added to the samples and controls. One set of samples and controls received 20 μL of anti-BoNT/A beads and the other set received 20 μL of anti-BoNT/B beads and 20 μL of anti-BoNT/F beads, which also attract BoNT/E. The deep well plate was capped and placed on a plate shaker for 1 h at the minimal speed necessary to keep the beads in solution. The samples were then processed in a KingFisher flex magnetic particle processor (Thermo Fisher Scientific, Waltham, MA, USA) using 2 M NaCl and PBST as washes as described earlier [18].

For quantitative analyses, sample volumes varied from 1 μL to 400 μL. The samples were analyzed in parallel with a negative control (described above) and a calibration curve as described previously [24].

### 4.4. Qualitative MS Analysis

The aqueous extract was removed from the beads, and all beads were reconstituted in 20 μL volumes consisting of reaction buffer (0.05 M Hepes (pH 7.3), 25 mM dithiothreitol, and 20 μM ZnCl_2_) and peptide substrate specific for the antibody extract (SubA, or a mixture of SubB, SubE, and SubF, respectively). Anti-BoNT/A beads were reconstituted in a mixture of 90% reaction buffer and 10% peptide substrate (final concentration of 50 μM). Anti-BoNT/F beads were reconstituted in a mixture of 75% reaction buffer, 10% Sub B (final concentration of 50 μM), 10% Sub F (final concentration of 50 μM), and 5% Sub E (final concentration of 25 μM). 

All samples were incubated at 37 °C for 4 h with no agitation. A 2 μL aliquot of each reaction supernatant was mixed with 18 μL of matrix solution consisting of α-cyano-4-hydroxy cinnamic acid (CHCA) at 5 mg/mL in 50% acetonitrile, 0.1% trifluoroacetic acid (TFA), and 1 mM ammonium citrate. A 0.5 μL aliquot of this mixture was pipetted onto one spot of a 384-spot matrix-assisted laser desorption/ionization (MALDI) plate (Applied Biosystems, Framingham, MA, USA). Mass spectra of each spot were obtained by scanning from *m/z* 900 to 5500 in MS-positive ion reflector mode on an Applied Biosystems 5800 Proteomics Analyzer (Framingham, MA, USA). The instrument uses an Nd-YAG laser at 355 nm, and each spectrum is an average of 2400 laser shots.

### 4.5. Quantitative MS Analysis

Two-stage Endopep-MS quantification consisting of singleplex analysis for BoNT/A and multiplex analysis for BoNT/B, /E, and F was performed as described previously [24], with the following changes: the toxins used for calibration were dichain BoNT at 1 μg/mL rather than BoNT complex at 1 mg/mL, and the sample volumes for this study varied as described in Section 4.3.

### 4.6. MS/MS Analysis

The BoNT immunoaffinity captured from the samples was digested with trypsin and chymotrypsin with a previously described protocol [28]. Peptides from the toxin were identified by LC-MS/MS with database searching as described previously [28].
